# GDEMM2024: Global Digital Elevation Merged Model 2024 for surface, bedrock, ice thickness, and land-type masks

**DOI:** 10.1038/s41597-024-03920-x

**Published:** 2024-10-04

**Authors:** E. Sinem Ince, Oleh Abrykosov, Christoph Förste

**Affiliations:** 1 Helmholtz-Centre GFZ German Research Centre for Geosciences, Global Geomonitoring and Gravity Field, Potsdam, Germany; 2 Helmholtz-Centre GFZ German Research Centre for Geosciences, Global Geomonitoring and Gravity Field, Oberpfaffenhofen, Germany

**Keywords:** Geophysics, Structural geology

## Abstract

Various research topics in geosciences such as gravity modelling, terrain correction and ocean circulation, require high resolution and accuracy global elevations for land topography, bathymetry, and ice thickness that refer to a consistent vertical datum. Unfortunately, most of the existing DEMs do not provide such solutions for Earth relief layers with the same resolution globally. To overcome this deficiency, we merged various DEMs published in the recent years and compiled an up-to-date global solution. We provide 30 arcsecond grid suite for relief layers and land-type masks which have been substantially improved w.r.t. the grids in literature. The quality of the merged surface elevation is assessed against the GNSS heights at about globally distributed 22000 stations. The merged surface model shows a reduction in standard deviation of a factor of three compared to other commonly used DEMs. Other evaluations are performed over land-ice and oceans which supports the advancement of GDEMM2024. The improvements are due to the accuracy and coverage of the original input data, updated land-type masks and merging methodology.

## Background & Summary

**Fig. 1:**
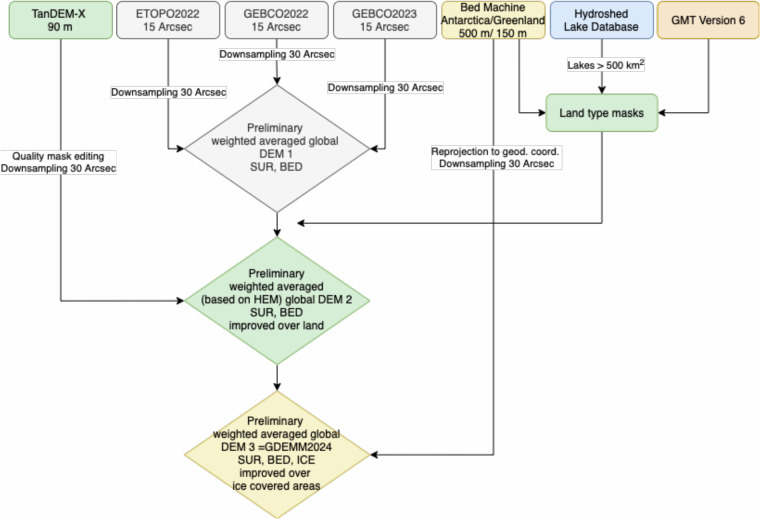
Construction of GDEMM2024.

Topography and bathymetry are essential in various Earth science disciplines such as gravity field modelling of the Earth^1^, crustal modelling^2^, and ocean floor modelling^3^. With modern technologies such as Synthetic Aperture Radar (SAR), echo sounding, radar altimetry and airborne radar measurements, we are now able to measure over inaccessible areas and can distinguish the elevation of different layers, such as sub-ice topography from the surface of the ice as well as the elevation of the ocean floor (bathymetry). Some missions are dedicated to provide high-accuracy elevation over dry land^4^, whereas some others are designed to retrieve depths of oceans and lakes^5^, or thickness of ice sheets^6^. Many applications require a global representation of topography, bathymetry, and ice thickness. In our study, as summarised in Fig. 1, we merge various data sets and measurements taking into account improved land-type masks for ice covered areas and lakes that were assumed as dry-land in previous models.

In order to take advantage of TanDEM-X and BedMachine data, we assign them more weights in the dedicated areas (mountainous land and ice-covered areas). Therefore, two preliminary models are computed instead of averaging all the models at a single step. HEMs (Height Error Maps) are used for weighted averaging of TanDEM-X. Land type masks are arranged using GMT V6, BedMachine over Antarctica and Greenland and HydroShed database over lakes globally. As a result of our study, the Earth relief is represented in different layers^7,8^.

1. The physical Earth surface (bedrock elevation over ice free continents, zero for ocean and ice surface over Greenland and Antarctica),
2. Bedrock (surface topography over continents, bathymetry over oceans, bedrock topography under grounded or floating ice; can be summarised as Earth’s relief without water and ice),
3. Ice thickness (ice-based topography over Greenland and Antarctica, zero over ice-free areas).

Earth without water masses, TBI (topography, bathymetry, ice) is also useful for different geophysical applications and visualization purposes and is represented by merging layers 2 and 3.

The 1 arc minute resolution Earth2014^7^, Earth’s relief model is widely used in geodetic and geophysical applications such as forward gravity modelling^9^, ultra-high resolution gravity field modelling^10^, heat flow mapping^11^, and crustal thickness modelling^12^. Since that model, new data sets became available which substantially improve the elevation of surface, bedrock, and ice for higher resolution global coverage, to mask lands and oceans more precisely that also helps distinguishing shorelines better including essential lakes globally. ETOPO2022 is a more recent model but it only includes the previous releases of shipborne measurements over oceans and airborne radar measurements over ice covered areas.

It is worth mentioning that, the majority of the above-mentioned applications require also the information on density^13–15^. The importance of the density in gravimetric forward modelling has been elaborated in various studies^16–18^. The merged model GDEMM2024 presented in this study is further used in forward modelling studies where the accuracy of the density estimations^19,20^ is also introduced. The results will be presented in another contribution.

## Methods

Our goal is to provide a Global Digital Elevation Merged Model (GDEMM) that is derived from the most recent topography and bathymetry models over the globe including ETOPO2022, GEBCO2022 and GEBCO2023, TanDEM-X and BedMachine grids which are of different spatial coverage, resolution, and accuracy. The data used in the merging procedure are available from open data archives. Based on these models, we develop a set of 30 arcsec (see Fig. 1) and 1 arcmin GDEMM2024^21^ global Earth relief layer grids for surface, bedrock, and ice thickness that are made publicly available via 10.5880/GFZ.1.2.2024.002. In this article, we describe the data sets, the methods used for merging and the evaluation with independently measured topographic heights.

**Fig. 2:**
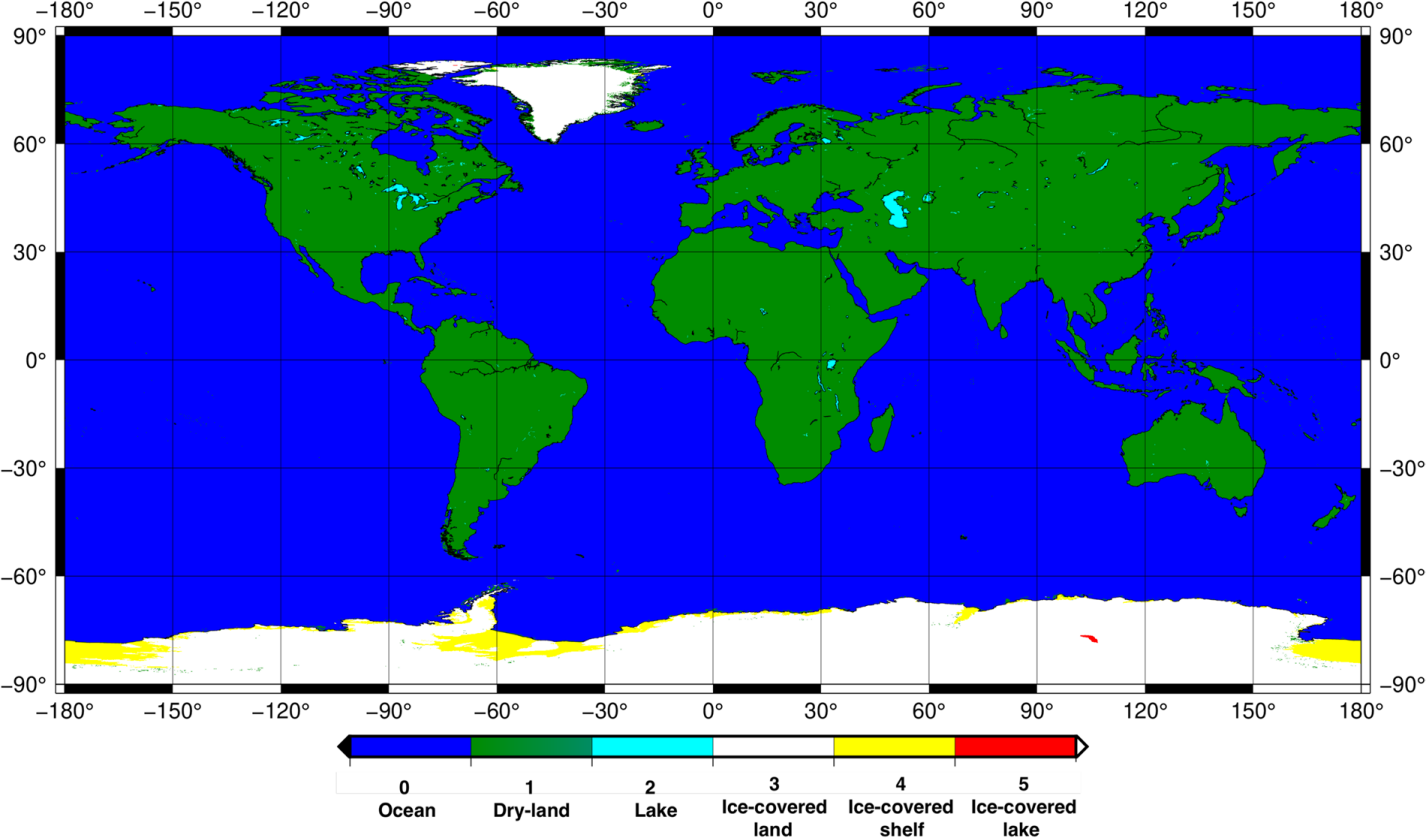
Spatial distribution of the masks used in the merging procedure for GDEMM2024: dry land (green), oceans (blue), lakes larger than 500 km^2^ (cyan), ice-covered land (white), ice-covered shelf (yellow) and ice-covered lakes (red).

As summarised in Fig. 1, GDEMM2024 merges seven data sets of topography, bathymetry and ice thickness that are improved with respect to data sets used in Earth2014 and ETOPO2022. The merging is performed using land-type masks retrieved from GMT (Generic Mapping Tools), BedMachine grids and HydroSHEDS archive as detailed in Table 1. As shown in Fig. 2, dry land, oceans, lakes larger than 500 km^2^, ice-covered land, ice-covered shelf and ice-covered lakes are included in the land-type masking scheme. The inclusion of the lakes is an important feature for applications such as forward gravity modelling or terrain correction since assigning the correct density for lakes may introduce substantial differences in the final product. Moreover, sub-ice lakes such as Lake Vostok (red coloured in Antarctica in Fig. 2) are also identified in the land-type mask. The importance of assigning correct laterally varying density estimations including the effect of depth dependent density variations are already highlighted for marine and land applications in literature^16–20^.

**Table 1:** GDEMM2024 relief layers, their characteristics, masks used, and validity check criteria used in the mask arrangement.

Earth relief layer	Description	Oceans	Dry Land	Lakes > 500 km^2^	Ice-covered land	Ice-covered shelf	Ice-covered lake
		Mask = 0 (GMT)	Mask = 1 (GMT)	Mask = 2 (GLOBathy)	Mask = 3 (BedMachine)	Mask = 4 (BedMachine)	Mask = 5 (BedMachine)
**SUR** *(ETOPO2022, GEBCO2022, GEBCO2023, TanDEM-X, BedMachines)*	Earth’s surface (Actual physical surface)	0	Topography	Surface (w.r.t. MSL)	Surface	Surface	Surface
**BED** *(ETOPO2022, GEBCO2022, GEBCO2023,BedMachines)*	Earth’s bedrock (Surface without water and ice)	Bedrock	Topography	Bedrock	Bedrock	Bedrock	Bedrock
**ICE** *(BedMachines)*	Earth’s ice over land and shelf (Ice thickness over ice covered areas)	0	0	0	Ice thickness	Ice thickness	Ice thickness
**TBI (BED + ICE)**	Earth’s topography, bedrock, and ice (Earth without water)	Bedrock	Topography	Bedrock	Surface	Surface	Surface
**Validity check**		ice = 0 surface = 0 bedrock< surface	ice = 0 surface = bedrock	ice = 0 bedrock < = surface	ice > 0 bedrock = surface-ice	ice > 0 bedrock < surface-ice	ice > 0 bedrock < surface-ice

The seven data sets used in the construction of GDEMM2024 are as follows:

The **TanDEM-X 90 m elevation data** (TerraSAR-X add-on for Digital Elevation Measurements) uses SAR interferometry to create a precise 3D map of the Earth’s land surfaces homogeneously with an unprecedented accuracy^4,22^. We use the non-edited TanDEM-X data that covers all Earth’s landmasses with a reduced pixel spacing of 3 arcsec approximately corresponding to 90 m at the equator. We post-processed the 19389 1 × 1 degree georeferenced TIFs by applying quality indication flags WAM (Water Indication Mask) due to temporal decorrelation and low backscattering, LSM (Layover and Shadow Mask), COM (Consistency Mask), and COV (Coverage Map), as recommended for the non-edited version^22^. Such masks help eliminating artifacts, outliers, noisy and invalid areas, but introduce void areas as a result that need to be filled-in. The ellipsoidal heights are converted to the heights above the geoid and down-sampling to a 30 arcsec grid is performed by using the Height Error Map (HEM) provided together with TanDEM-X grids. The error sources of the HEM include among others high-relief terrain, forested areas, snow and ice, and sandy deserts^4^. The TanDEM-X data are highly accurate which have been tested against about three million globally distributed GPS points and 23,000 GPS on Benchmarks scattered over various land types^22^ showed that the absolute vertical mean error of the TanDEM-X surface elevation is smaller than ± 0.20 m and has an RMS (Root Mean Square) smaller than 1.4 m. In our study, for down-sampling each node in the 30 arcsec grid, weighted averages are computed based on the 90 m resolution HEMs. After down-sampling, data gaps that exist because of the quality masking have been filled-in by using linear interpolation in the East-West and North-South direction separately. The interpolation is applied first for a predefined length starting from cell size 30 arc-sec. In the next step, this length of gap interval is increased which causes smoothened values over large gaps. The interpolation is performed for dry land and ice-covered areas separately. An alternative would be filling these areas using other DEMs available.

The **GEBCO_2022** data^23^ (General Bathymetric Chart of the Oceans, GEBCO_2022) is a global terrain model over land and ocean. The grid includes the SRTM15 + base grid v2.4^24^ between latitudes 50° S and 60° N, augmented with the gridded bathymetric data sets developed by the Seabed 2030 Regional Centers^23^. Beyond areas north of 60° N, land data are mostly based on the Global Multi-resolution Terrain Elevation Data 2010^25^ (GMTED2010). We used two georeferenced TIFs for surface topography (land and ice surface elevation) and sub-ice topography (bedrock), provided in 15 arcsec resolution and down-sampled them to 30 arcsec resolution for our purpose. The grids incorporate ice-surface elevation and under-ice topography/bathymetry from IceBridge BedMachine Greenland, Version 4.6^26^ and MEaSUREs BedMachine Antarctica, Version 2^27^.

The **GEBCO_2023** grid^28^ is also a continuous, global terrain model for ocean and land provided with a spatial resolution of 15 arcsec. The grid relies on the SRTM15 + v2.5.5^24^ data between latitudes 50° S and 60° N which is augmented with the gridded bathymetric data that has been updated and improved w.r.t. the 2022 version (https://www.gebco.net/about_us/acknowledgements/our_data_contributors/). The rest of the data used over land is largely based on the same data as GEBCO_2022. Validation studies performed over different test areas in shallow water suggest an RMS of 3-5 meter compared to ICESAT-2 results^29^. The GEBCO_2023 grids are also available in two versions for land and ice surface elevation and sub-ice topography and given in ellipsoidal heights w.r.t. the WGS84 ellipsoid. We used the georeferenced TIF of 15 arcsec resolution and down-sampled it to 30 arcsec resolution grids. Same BedMachine grids are used in the construction of GEBCO_2023 as in GEBCO_2022, which however have been updated thereafter. Therefore, we utilize instead the most recent BedMachine grids as described below. The reason why we included both GEBCO_2022 and GEBCO_2023 is due to the missing information in the related literature concerning the new measurements incorporated, reliability and differences of the two models. We expect that averaging the GEBCO series reduces the larger present errors.

**ETOPO 2022**^30^ is the most recently released NOAA Earth TOPOgraphy data set. It consists of various DEMs and bathymetry data including GEBCO_2022 and previous versions of BedMachine^26,27^, regional DEMs from NOAA, and Copernicus DEM 30 m which is acquired through TanDEM-X. It is a global, seamless, topographic and bathymetric bare-earth elevation data set of 15 arcsec resolution validated against ICESAT-2 elevation data. Additional files are released to provide bedrock elevation under the ice sheets over Greenland and Antarctica. The ETOPO 2022 data refer to WGS84 in the horizontal coordinates and to the Earth Gravitational Model 2008 (EGM2008^31^) geoid surface for the height component. In this study, we used the 15 arcsec grids and down-sampled it into 30 arcsec for our purposes.

**BedMachine v5 Greenland:** These data contain bedrock topography and bathymetry information over Greenland that is derived based on mass conservation and ocean bathymetry data from various sources. Bed elevation is provided relative to the EIGEN-6C4 geoid^32^. The coverage of the final product is between 60–90° N and 80° W - 10° E and its spatial resolution is 150 m, whereas the resolution of the input data varies between 150 m and 5 km. Bedrock topography and ice thickness data are mostly collected by airborne radar which is inadequate over coastal areas. Various data sets are merged for the purpose of a high-quality bed topography and fjord bathymetry map of Greenland^26,33^.

**BedMachine v3 Antarctica: **These data contain a bed topography and bathymetry map of Antarctica that is derived via subtracting the ice thickness from the surface elevation based on the Reference Elevation Model of Antarctica (REMA^34^). The coverage of the final product is south of 70° S and its spatial resolution is 500 m. Ice thicknesses were derived via mass conservation, streamline diffusion and other methods^27,35^. In most parts, the ice thicknesses were derived from airborne ice penetrating radar systems. The ice thickness and ice surface elevations refer to heights above the EIGEN-6C4 geoid.

**Lakes:** We incorporate depth information (height from bedrock to water surface) for lakes via utilising the GLOBathy data set (Global Lakes Bathymetry^36^). The GLOBathy database includes information on 1,427,688 lakes and reservoirs and is published in terms of georeferenced TIFs for depths and shapefiles for corresponding polygons (https://www.hydrosheds.org/products/hydrolakes). While incorporating the lakes in the relief grids, we used the averaged surface elevations and depth information provided for each lake. A homogenous relief layer is created via referring all the lake surfaces to the EIGEN-6C4 geoid. Lake attributes containing surface areas and surface elevations (above MSL) have been converted from HydroSHEDS polygons into an ASCII file and sorted based on their surface areas. In our merging strategy, we only included the lakes larger than 500 km^2^ as shown in Fig. 2. The corresponding areas inside the polygons in the surface elevation from the merged DEMs are replaced with the values derived from the GLOBathy^36^ data set and the surface of lake and bedrock (depths) are included in the relief grid.

**Mask grids:** Land-type mask grids are important in the preparation of the merged relief components. We have retrieved available land-type masks from the BedMachine grids over Greenland and Antarctica and from the GMT Version 6^37^ for the rest of the globe (see Table 1). The BedMachine masks have been reprojected from Polar Stereographic Projections (rectangular coordinates) into WGS84 geodetic coordinates. Moreover, we used the shape files provided by the HydroSHEDS database to create masks for lakes that are larger than 500 km^2^. In a sequential manner, the masks for lakes, ice-covered land, ice-covered shelf, ice-covered lakes, ocean and dry-land areas are merged as summarised in Table 1 and shown in Fig. 2. The use of improved masks over ice covered areas and lakes compared to Earth2014 is particularly important in terms of avoiding edge effects over different land-types. Masks have been organized and used in the merging procedure of the grid files to define the upper and lower boundaries of the relief compartments. The validity of the masking schemes was quality- checked as given in Table 1.

The merging procedure presented in Fig. 1 has been conducted in a strategic sequence based on the accuracy assessments of the data sets in the following way.

- The DEM combination starts by calculation of preliminary grids for surface and bedrock from ETOPO2022, GEBCO_2022, and GEBCO_2023 by means of weighted average grids w.r.t. the arithmetic average of the three.
- A preliminary geoid grid corresponding to these averaged grids for surface and bedrock has been taken as down-sampled (30 arcsec) geoid grid of ETOPO2022 which is based on EGM2008. The tide system of this reference geoid was not listed. After running some comparisons, tide-free properties have been assigned.
  The surface and bedrock elevations provided by each of the three DEMs as well as their surface minus bedrock differences were investigated. All three DEMs have negative differences reaching up to −1061 m (see Table 1 in Supplementary Document). In other words, the bedrock elevation provided by the models is higher than the surface elevation over certain areas. These values should represent the ice thickness and therefore should be positive. The negative values indicate larger errors for the measured ice thickness in these models which is solved by step e) below.
- A preliminary land-type mask with the same spatial resolution has been created as described above. The global lakes were incorporated in this preliminary version (see Fig. 2).
- The averaged preliminary surface grid from a) was merged with the TanDEM-X surface grid via a weighting scheme which leads to improvements over mountainous terrain types. Inconsistencies among the background geoids have been eliminated by referring all grids to EIGEN-6C4 geoid and applying tide-free system globally.
- Due to the shortcomings mentioned in step b), we incorporate the BedMachine grids in the polar regions masked in step c) by replacing cells of all five (surface, bedrock, ice, geoid, mask) preliminary grids by the corresponding data from BedMachine. BedMachine v5 is used for Greenland, whereas Bedmachine v3 is used for Antarctica.

**Fig. 3:**
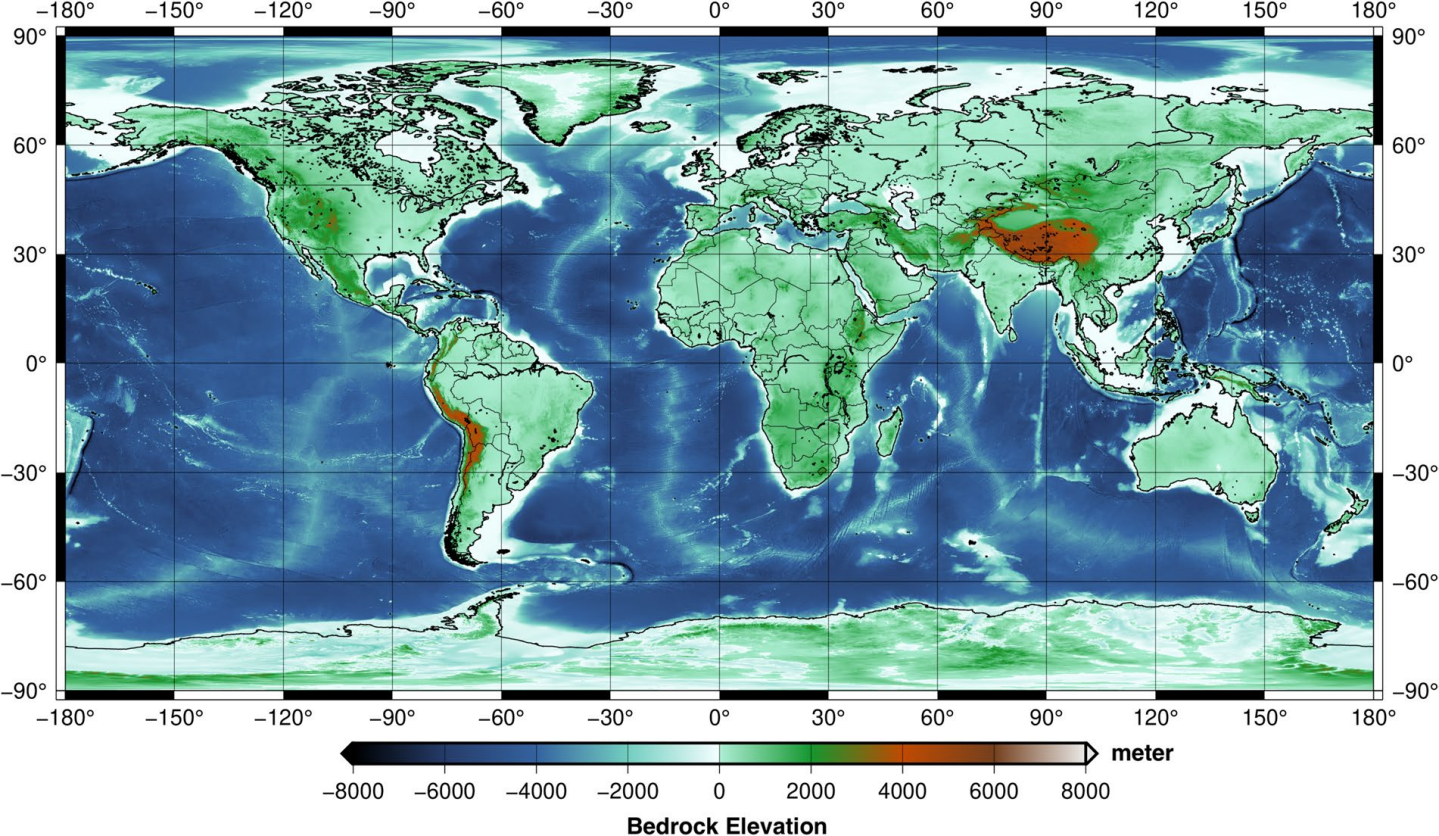
30 arcsec GDEMM2024 Bedrock grid in metres.

The final merged bedrock is shown in Fig. 3. The bedrock corresponds to surface of the topography over ice free land, bottom of bathymetry over oceans. Over ice covered areas, such as Greenland and Antarctica, bedrock surface would be retrieved via removing the ice thickness from the measured surface elevation.

**Fig. 4:**
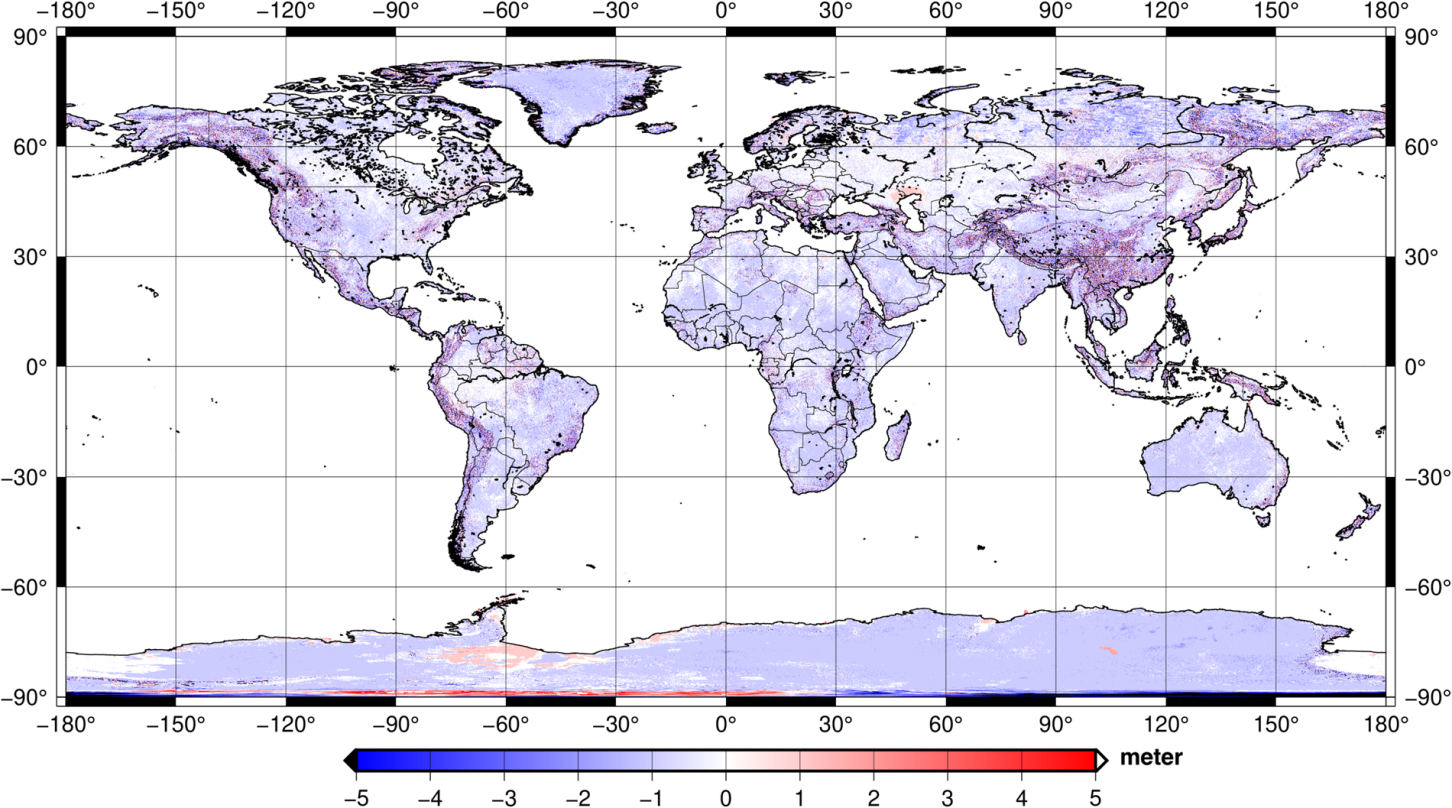
Global coverage of the contribution of TanDEM-X on the weighted combined solution of ETOPO2022, GEBCO 2022, and GEBCO2023 over dry-land and ice-covered areas for bedrock, in meter.

From literature, we can claim that most of the contribution coming from TanDEM-X is on areas of inaccessible terrain and mountainous regions. The differences between the bare-Earth and surface, in other words, Digital Elevation and Digital Surface Models, were neglected for our merged product. The contribution of TanDEM-X on the weighted combination of ETOPO2022, GEBCO_2022, and GEBCO_2023 over dry-land and ice-covered areas are shown in Fig. 4. The differences are concentrated in terrain with high topography, with the maximum and minimum values reaching about ±30 metres.

**Fig. 5:**
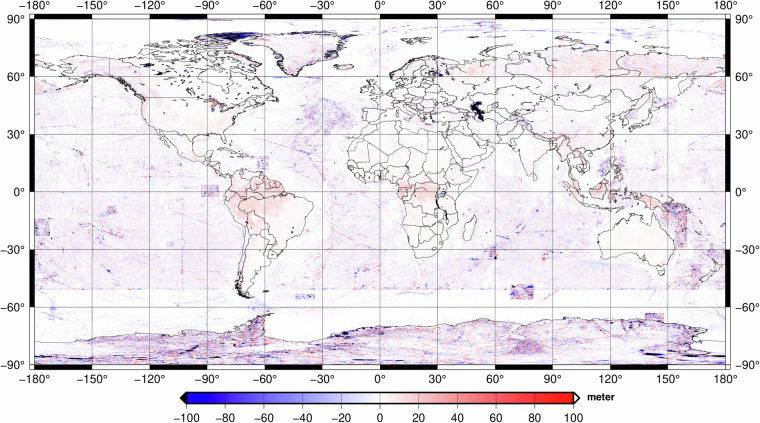
Differences between GDEMM2024 and ETOPO2022 bedrock grids, given in meter. GDEMM2024 includes both GEBCO2022 and GEBCO2023, whereas ETOPO2022 is based on GEBCO2022 grid.

As a further indication of improvement w.r.t. previous topography models, the differences between the GDEMM2024 and ETOPO2022 bedrock grids are shown in Fig. 5. The largest differences appear mostly over oceans, ice covered areas and mountainous regions. From literature, we know these improvements result from new shipborne measurements included in GEBCO_2023, and new versions of BedMachine grids used over Greenland and Antarctica and TanDEM-X data included, respectively.

The data sets used in the merged model are not free of errors and inclusion of new data sets and updates in the final merged product over the years will be essential. A review of the general DEM reconstruction and fusion methods used in the development of more comprehensive DEMs via using their complementary characteristics is investigated by others^38^. Our method explained in this section allows easy incorporation of updated DEMs and related products that serve most of the geodetic, geophysical and oceanographic applications by providing readily usable grids.

## Data Records

The content of the relief grids is represented w.r.t. the EIGEN-6C4 geoid in the WGS84 datum (EPSG code: 9055) and comprises the following 30 s georeferenced TIFs: GDEMM2024_XXX.30 s.tif, where XXX stands for SUR, BED, ICE TBI, LTM (Land Type Mask) and GEO (Geoid). SUR represents the topography over dry-land, the surface of the water bodies, and the ice sheets. BED represents again the topography over dry lands, the elevation of the bottom of ocean floor and lakes, and the sub-ice bedrock topography. ICE represents the ice thickness over ice covered areas in Greenland and Antarctica. Additionally, TBI (topography, bedrock, ice) grid incorporates the BED and ICE grids to provide Earth surface without the water.

**Fig. 6:**
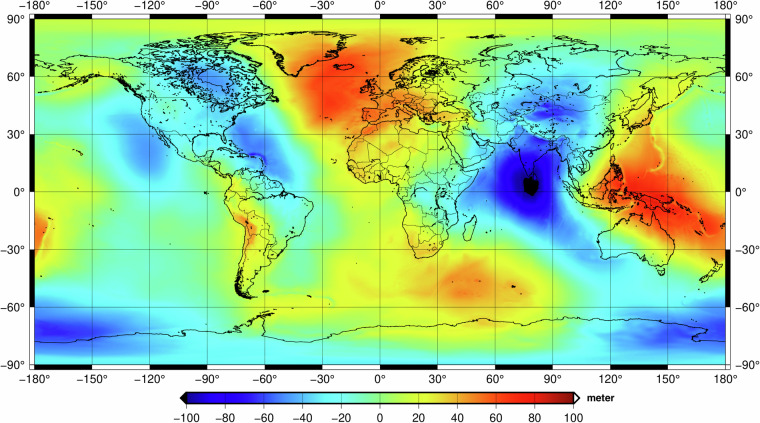
The EIGEN-6C4 geoid model that can be used to convert GDEMM2024 heights (w.r.t MSL) into ellipsoidal heights w.r.t WGS84 ellipsoid.

Statistics of the available 30 arcsec grids are summarised in Table 2. For convenience, 1 arcmin down-sampled grids are also included in the published dataset. The EIGEN-6C4 geoid grid, presented in Fig. 6, is provided to enable the transformation of the heights above the geoid, GDEMM heights, into ellipsoidal heights. Finally, a land-type mask is provided for distinguishing the land, ocean, inland lakes, and ice-covered areas (see Fig. 2). All the grids are available on 10.5880/GFZ.1.2.2024.002. For the direct use of GDEMM2024^21^ in calculations in the spectral domain such as harmonic synthesis in geodetic and geophysical applications^39^, the grids can be transformed into spherical harmonic coefficients which is not a trivial task.

**Table 2:** The statistics of the surface and bedrock elevations and ice thickness layers of the final 30 arcsec weighted relief layer grids of GDEMM2024, given in metres.

Relief layer	Max	Min	Mean	Rms	Std
Surface (SUR)	8387.00	−417.00	378.61	936.90	857.00
Bedrock (BED)	8387.00	−10792.00	−2128.81	3173.13	2553.07
Ice thickness (ICE) (Greenland and Antarctica)	4807.00	0.00	229.41	768.14	733.09
Topography/Bedrock/Ice (TBI)	8387.00	−10792.00	−1896.59	3264.61	2657.18

## Technical Validation

The GDEMM2024^21^ was evaluated against three different types of data:

1. The heights of more than 22000 GNSS stations on the surface that are downloaded from Nevada Geodetic Laboratory (NGL) (http://geodesy.unr.edu/index.php)^40^.
2. The along track ICESAT-2^41^ (Ice, Cloud and land Elevation Satellite-2) surface elevations collected by the Advanced Topographic Laser Altimeter System (ATLAS) instrument on board the ICESat-2 over land-ice area in Antarctica.
3. Bathymetry measurements along ship tracks^42^, 500 m grid resolution bathymetry model over Baltic Sea^43^ and GEBCO2024^44^ which has been released recently.

**Fig. 7:**
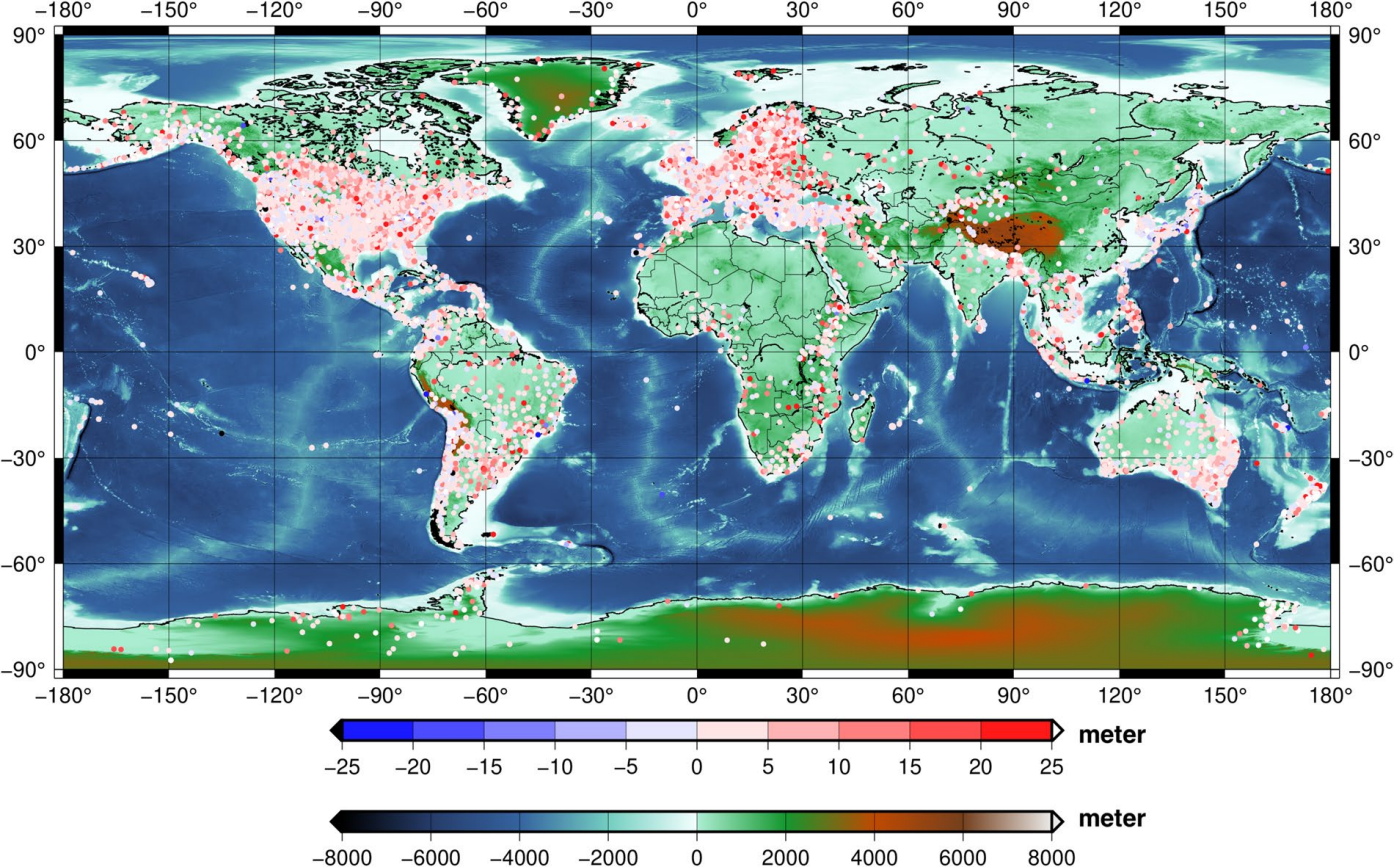
The GDEMM2024 TBI (Bedrock + Ice) displayed in the background together with the surface elevation differences to the NGL heights at 22122 stations.

The ellipsoidal heights of the NGL stations were transformed into the heights above the EIGEN-6C4 geoid and the GDEMM2024 elevations were interpolated to the NGL points and subtracted from those. The differences are shown in Fig. 7 at globally distributed GNSS stations with the GDEMM2024 surface grid in the background. Overall positive values stem from the positions of the stations that can be located metres above the surface of the Earth. It was not possible to find relevant metadata concerning the elevation of the benchmarks over the ground. Therefore, the ellipsoidal heights are taken as are and not corrected for the station elevation from the ground in our investigations. Moreover, some of the stations show unrealistic values such as HRR2, where the ellipsoidal heights reach lower than −900 metres. Such suspicious stations detected as blunders have been removed in our statistics.

In order to investigate the error w.r.t. increasing elevation, we categorized the comparisons for different terrain types, representing flat, moderate and mountainous areas by different elevation ranges as shown in Table 3. The same comparison is performed for ETOPO2022 and other elevation models for the entire dataset which are summarized in Table 4 and detailed in Tables 3–6 in the Supplementary Document. Comparison of point data with a large footprint of 30 arcsec cell size is erroneous due to the down-sampling. Therefore, the statistics presented here do not represent absolute vertical accuracy values, but supports relative comparisons of different terrain types and the models listed. Based on these analyses, GDEMM2024 outperforms the existing DEMs and shows increasing error for increasing topographical heights as expected.

**Table 3:** The differences of the GDEMM2024_TBI w.r.t. the elevations retrieved at the NGL GNSS stations (22122) over dry-land and ice, given in metres.

Terrain category range in metre	Number of stations	Max	Min	Mean	Rms	Std
[−407 – 0]	1370	50.33	−504.42	2.50	14.94	14.73
[0–200]	9537	199.81	−65.13	4.61	11.20	10.20
[200–600]	6057	219.96	−118.80	5.96	18.06	17.04
[600–1200]	2608	284.61	−169.31	8.45	27.44	26.10
[1200–2600]	2286	244.07	−568.34	11.78	37.03	35.11
[2600–5273]	264	302.44	−61.71	24.65	57.19	51.60

**Table 4:** The differences of GDEMM2024 and other DEMs w.r.t. the elevations retrieved at 22122 NGL stations, given in metres.

DEM	Max	Min	Mean	Rms	Std
GDEMM2024	302.44	−568.34	6.28	20.65	19.68
TanDEM-X	685.10	−568.34	7.31	26.85	25.84
ETOPO2022	2980.21	−583.34	9.48	65.69	65.00
GEBCO2022	2981.21	−569.34	8.63	65.69	65.13
GEBCO2023	2981.21	−569.34	8.99	65.49	64.87

**Fig. 8:**
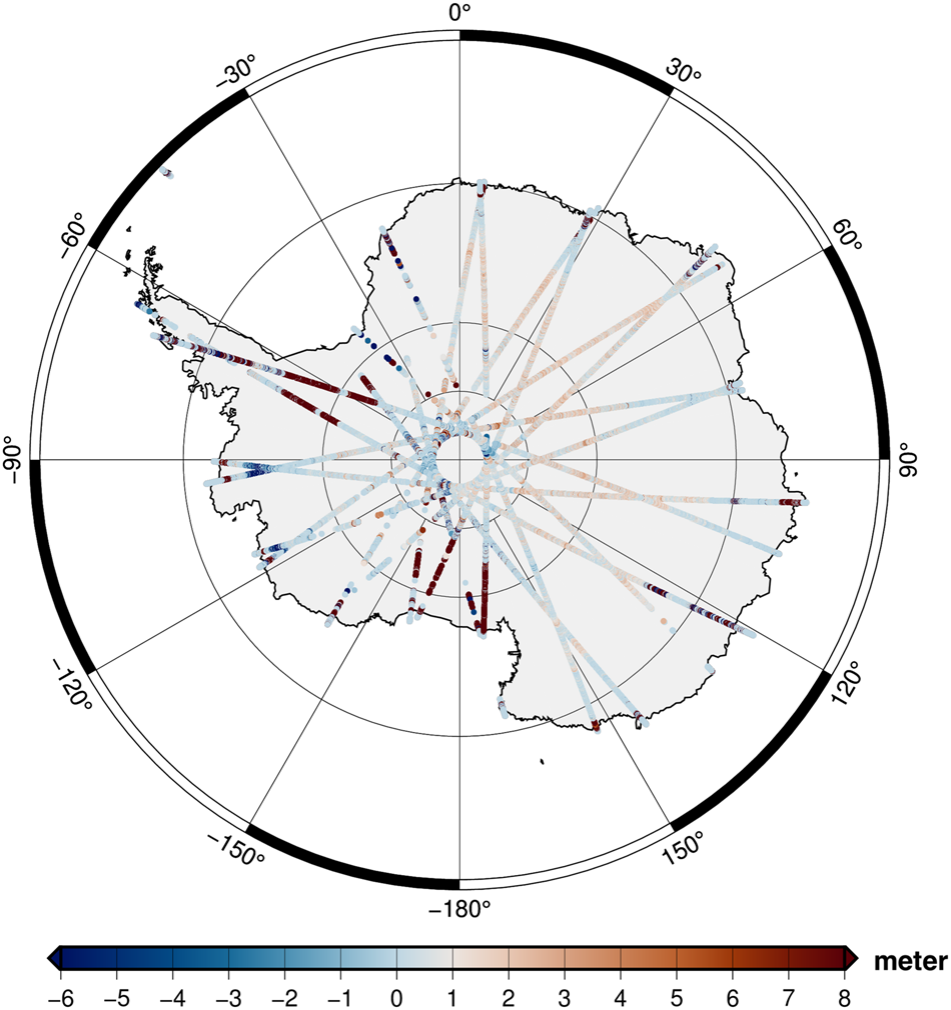
The differences of GDEMM2024 TBI w.r.t ICESAT-2 are shown for Antarctica.

For investigations over land-ice, the ICESAT-2 ATL06 data are downloaded from https://nsidc.org/data/data-access-tool/ATL06/versions/6. The datasets are given along orbit tracks and categorised as Level 3 A data. The land-ice surface heights are given w.r.t. WGS84 ellipsoid, ITRF2014 reference frame which are again transformed into the heights above EIGEN-6C4 geoid as done for NGL GNSS benchmarks. ICESAT-2 comparisons are performed along tracks collected over Antarctica without performing any further corrections. The tracks are dated between 16.03.2024 to 17.03.2024 which makes them independent new series for the evaluation of GDEMM2024. The differences of GDEMM2024 and ICESAT-2 heights are shown in Fig. 8 and summarised in Table 5 together with the statistics of other DEMs included in this study. The analyses show that all the DEMs included in this study are in about 30 m agreement with ICESAT-2 height measurements in terms of standard deviation which also indicates the adequacy of GDEMM2024 in this region compared to the other commonly used DEMs.

**Table 5:** The differences of GDEMM2024 and other DEMs w.r.t. the elevations retrieved at 2405990 ICESAT-2 points, given in metres.

DEM	Max	Min	Mean	Rms	Std
GDEMM2024	2011.06	−1468.03	2.15	30.41	30.33
TanDEM-X	2011.06	−1468.03	2.13	30.01	29.94
ETOPO2022	2013.06	−1521.26	3.67	27.68	27.43
GEBCO2022	2035.06	−1506.26	21.37	37.74	31.10
GEBCO2023	2084.07	−1507.26	21.34	38.34	31.83

The third evaluation is performed over the oceans using different datasets. Use of bathymetry data measured along ship tracks downloaded from http://data.utm.csic.es/set/sdg/20181116/ for such comparisons is very challenging due to potential high noise level included in the *in-situ* measurements where the mean value of the differences reaches to several hundreds of meters. This does not help to distinguish GDEMM2024 from other DEMS (see Table 7 in the Supplementary Document). Moreover, since GDEMM2024 has been merged based on GEBCO2022 and GEBCO2023 and most of such *in-situ* measurements are included in GEBCO series already, such analyses will not provide distinctive results despite the large differences. Alternatively, comparisons to a dedicated Baltic Sea bathymetry generated in 2013 (Baltic_Sea_Bathymetry_Database_v091.tif, https://metadata.helcom.fi/geonetwork/srv/api/records/8b46e4c7-f911-44ab-89e6-2c8b8d9fa2c0) indicate an agreement about 7 m in terms of standard deviation as shown in Fig. 1 in the Supplementary Document. It is worth noting that GDEMM2024 is not completely independent from this grid data either. Finally, GEBCO2024 released recently is used in our comparisons which indicates better agreement with GDEMM2024 than other models included in the study. The agreement is investigated in terms of the differences of the grid files published openly (https://www.gebco.net/data_and_products/gridded_bathymetry_data/). The differences are in the range of several tens of meters and presented in Table 6. Such differences indicate the challenging nature of ocean floor modelling, the high noise level involved in the datasets as well as the improved coverage over oceans.

**Table 6:** The differences of GDEMM2024, ETOPO2022 and GEBCO series w.r.t each other over oceans.

DEM	Max	Min	Mean	Rms
GDEMM2024_min_ETOPO2022	2054.00	−3314.00	−0.77	15.13
GDEMM2024_min_GEBCO2022	2054.00	−1820.00	−0.57	13.30
GDEMM2024_min_GEBCO2023	3760.00	−4258.00	−0.09	24.61
ETOPO2022_min_GEBCO2022	4867.00	−2787.00	0.20	15.19
ETOPO2022_min_GEBCO2023	5580.00	−6312.00	0.68	37.46
GEBCO2022_min_GEBCO2023	5580.00	−6312.00	0.48	35.21
GDEMM2024_min_GEBCO2024	5666.00	−4772.00	−1.78	58.97
ETOPO2022_min_GEBCO2024	5663.00	−4772.00	−1.01	62.91
GEBCO2022_min_GEBCO2024	5663.00	−4857.00	−1.21	62.07
GEBCO2023_min_GEBCO2024	6385.00	−5580.00	−1.69	59.10

The reason for making such comparisons is not to answer which model is the best, but to show that dedicated merging steps together with inclusion of the most recently available datasets result in better accuracy DEM, GDEMM2024 than the individual ones included in its development. From our GNSS height comparisons, we can claim that, at a global scale GDEMM2024 shows an improvement of about a factor of three compared to the other commonly used models, in terms of standard deviation.

## Usage Notes

In this study, we produced a readily usable global merged digital elevation model that consists of surface and bedrock elevation, ice thickness, and land-type mask grids via merging various DEM sources. Forward gravity modelling for the development of ultra-high resolution global gravity field models, 3D gravity field modelling, terrain correction, crustal modelling, ocean floor, as well as flood modelling studies are among the applications of such suite of grids.

The GDEMM2024 has an advantage over land and ice-covered areas compared to currently used DEMs due to the inclusion of the most recently available surface measurements from TanDEM-X, ice thickness information from the BedMachine versions v5 over Greenland and v3 in Antarctica, global lake bathymetry, and improved land-type masks of ice-covered areas, shorelines, and lakes. We established a methodology that follows a strategic merging scheme based on the accuracy of the included datasets. Our validation studies of GDEMM2024 w.r.t. independent elevation measurements at about 20000 NGL GNSS stations suggest an error of smaller than 20 m which is about three times better than the previously available, widely used DEMs. Earth relief models are crucial not only in high resolution gravity forward modelling, but also in validation of synthetic datasets or satellite-derived observations in global gravity field modelling, calculation of gravity values over regions lacking ground observations, and calculation of complete Bouguer gravity anomalies and crustal modelling studies where the terrain correction should be introduced correctly based on the high-resolution surface, bedrock and ice thickness grids provided as a result of this study.

### Depositing your data to an appropriate repository

 The GDEMM2024 grids provided in 30 arcsec and 1 arcmin resolution are available on 10.5880/GFZ.1.2.2024.002 freely. The spherical harmonic coefficients of the relief layers can be made available upon request.

## Supplementary information

Supplementary_Ince_et_al_revised

## Data Availability

There is no ready to use custom code developed for the creation of GDEMM2024. Existing packages from GDAL v3.9 and GMT v6.4, such as gdalwarp (for reprojecting and downsampling), gdal_translate (for grid transformation from one format to another), gmt grdmath (for other operations) are used.
