# Supplementary material for: GDEMM2024: Global Digital Elevation Merged Model 2024 for surface, bedrock, ice thickness, and land-type masks
Source: Sci Data. 2024 Oct 4;11:1087. doi: 10.1038/s41597-024-03920-x (PMC11452699; doi:10.1038/s41597-024-03920-x)
Supplement: Supplementary file 1 — Supplementary_Ince_et_al_revised [file 41597_2024_3920_MOESM1_ESM.pdf]

Supplementary document to

GDEMM2024: Global Digital Elevation Merged Model 2024 for surface, bedrock, ice thickness, and land-type masks

Authors

E. Sinem Ince[1], Oleh Abrykosov[2], Christoph Förste[1]

Affiliations

1. Helmholtz-Centre GFZ German Research Centre for Geosciences, Global Geomonitoring and Gravity Field, Potsdam
2. Helmholtz-Centre GFZ German Research Centre for Geosciences, Global Geomonitoring and Gravity Field, Oberpfaffenhofen

corresponding author(s): E. Sinem Ince (elmas.sinem.ince@gfz-potsdam.de)

Table of Content

Table S1: Statistics of relief layers differences, ETOPO_2022, GEBCO_2022, and GEBCO_2023 in metres.

Table S2: Table2: Statistics of the three DEM, ETOPO2022, GEBCO_2022, and GEBCO_2023 differences in metres.

Table S3: Differences of TanDEM_X elevation grid and the elevations retrieved at 22122 NGL stations categorized over different terrain categories, in metres.

Table S4: Differences of GEBCO_2022 elevation grid and the elevations retrieved at 22122 NGL stations categorized over different terrain categories, in metres.

Table S5: Differences of GEBCO_2023 elevation grid and the elevations retrieved at 22122 NGL stations categorized over different terrain categories, in metres.

Table S6: Differences of ETOPO_2022 elevation grid and the elevations retrieved at 22122 NGL stations categorized over different terrain categories, in metres.

Table S7: Bathymetry comparisons between GDEMM2024_Bed and ORCA cruise measurements over the ocean along ship track

Fig S1: Comparisons of GDEMM2024 with 500 m grid resolution Baltic_Sea_Bathymetry_Database_v091 over the Baltic Sea.

Supplementary Document to

GDEMM2024: Global Digital Elevation Merged Model 2024 for surface, bedrock, ice thickness and land-type masks

SUR represents the topography over dry-land, the surface of the water bodies, and the ice sheets. BED represents again the topography over dry lands, the elevation of the bottom of ocean floor and lakes, and the sub-ice bedrock topography. ICE represents the ice thickness over ice covered areas in Greenland and Antarctica. Additionally, topography/bedrock/ice (TBI) grid incorporates the BED and ICE grids to provide Earth surface without the water.

Table1: Statistics of relief layer differences, ETOPO_2022, GEBCO_2022, and GEBCO_2023 in metres. The information is used to distinguish the areas of problematic ice thickness information existing in ETOPO2022, GEBCO_2022, and GEBCO_2023.

Relief layer	Max	Min	Mean	Rms
etopo22_sur_30s	8353.00	-10775.00	-1897.16	3263.71
gebco22_sur_30s	8437.00	-10919.00	-1898.69	3262.51
gebco23_sur_30s	8437.00	-10916.00	-1899.03	3262.93
etopo22_bed_30s	8353.00	-10775.00	-2127.96	3172.56
gebco22_bed_30s	8437.00	-10919.00	-2127.92	3172.78
gebco23_bed_30s	8437.00	-10916.00	-2128.26	3173.21
etopo22_sur-bed	4899.00	**-1061.00**	230.81	768.08
gebco22_sur-bed	4873.00	**-639.00**	229.23	762.92
gebco23_sur-bed	4874.00	**-622.00**	229.23	762.92

Table2: Statistics of the three DEM, ETOPO2022, GEBCO_2022, and GEBCO_2023 differences in metres.

Relief layer	Max	Min	Mean	Rms
gebco22-gebco23 (sur)	5580.00	-6312.00	0.34	28.83
gebco22-etopo22 (sur)	3098.00	-4867.00	-1.53	19.65
gebco23-etopo22 (sur)	312.00	-5580.00	-1.87	34.16
gebco22-gebco23 (bed)	5580.00	-6312.00	0.34	28.82
gebco22-etopo22 (bed)	3098.00	-4867.00	0.04	23.85
gebco23-etopo22 (bed)	6312.00	-5580.00	-0.29	36.73

Table 3: Differences of TanDEM_X elevation grid and the elevations retrieved at 22122 NGL benchmarks categorized over different terrain categories, in metres.

Terrain type Range in metre	Number of stations	Max	Min	Mean	Rms	Std
All	22122	685.1	-568.34	7.31	26.85	25.84
[-407 – 5273]	1370	444.91	-504.42	3.81	27.43	27.16
[0 – 200]	9537	464.60	-64.30	5.94	24.41	23.67
[200 – 600]	6057	685.10	-118.79	6.49	21.01	19.99
[600 – 1200]	2608	284.61	-169.31	9.01	29.32	26.85
[1200 – 2600]	2286	391.32	-568.34	13.09	39.48	37.25
[2600 – 5273]	264	302.44	-61.71	26.83	58.66	52.17

Table 4: Differences of GEBCO_2022 elevation grid and the elevations retrieved at 22122 NGL benchmarks categorized over different terrain categories, in metres.

Terrain type Range in metre	Number of stations	Max	Min	Mean	Rms	Std
All	22122	2981.21	-569.34	8.63	65.69	65.13
[-407 – 5273]	1370	329.23	-505.42	2.57	19.83	19.66
[0 – 200]	9537	564.94	-538.27	5.07	19.54	18.87
[200 – 600]	6057	851.70	-221.79	6.62	22.96	21.99
[600 – 1200]	2608	991.59	-170.31	11.03	41.61	40.12
[1200 – 2600]	2286	2969.75	-569.34	21.13	129.89	128.16
[2600 – 5273]	264	2981.21	-63.71	83.06	412.94	404.50

Table 5: Differences of GEBCO_2023 elevation models and the elevations retrieved at 22122 NGL benchmarks categorized over different terrain categories, in metres.

Terrain type Range in metre	Number of stations	Max	Min	Mean	Rms	Std
All	22122	2981.21	-569.34	8.99	65.49	64.87
[-407 – 5273]	1370	327.23	-505.42	3.43	20.15	19.86
[0 – 200]	9537	564.94	-74.30	5.65	19.73	18.91
[200 – 600]	6057	815.70	-221.79	6.93	24.61	23.61
[600 – 1200]	2608	516.76	-170.31	10.25	34.68	33.13
[1200 – 2600]	2286	2969.75	-569.34	21.71	130.26	128.44
[2600 – 5273]	264	2981.21	-63.71	83.42	413.00	404.48

Table 6: Differences of ETOPO2022 elevation models and the elevations retrieved at 22122 NGL benchmarks categorized over different terrain categories, in metres.

Terrain type Range in metre	Number of stations	Max	Min	Mean	Rms	Std
All	22122	2980.21	-583.34	9.48	65.69	65.00
[-407 – 5273]	1370	50.91	-501.42	3.70	15.46	15.01
[0 – 200]	9537	564.94	-86.76	6.02	19.16	18.18
[200 – 600]	6057	799.70	-76.65	7.24	22.38	21.18
[600 – 1200]	2608	377.30	-168.31	11.00	33.86	32.02
[1200 – 2600]	2286	2969.75	-583.34	22.96	133.14	131.14
[2600 – 5273]	264	2980.21	-55.71	83.66	413.20	404.64

Table 7: Bathymetry comparisons between GDEMM2024_Bed and ORCA cruise measurements over the ocean along ship track at 37336 points. Values within parenthesis are retrieved after removing 5% of the points that are exposed to large noise level.

DEM	Max	Min	Mean	Rms	Std
**GDEMM2024**	5103.04<br>(787.24)	-4913.36<br>(-499.56)	121.32<br>(11.258)	673.47<br>(105.00)	662.46<br>(104.39)
**ETOPO2022**	5130.66<br>(789.94)	-4913.36<br>(-495.56)	126.25<br>(11.09)	692.59<br>(105.78)	680.98<br>(105.20)
**GEBCO2022**	5130.66<br>(777.54)	-4913.36<br>(-495.56)	125.85<br>(10.735)	693.00<br>(104.59)	681.47<br>(104.04)
**GEBCO2023**	5130.04<br>(769.94)	-4913.36<br>(-512.56)	125.23<br>(10.48)	690.16<br>(100.91)	678.70<br>(100.34)

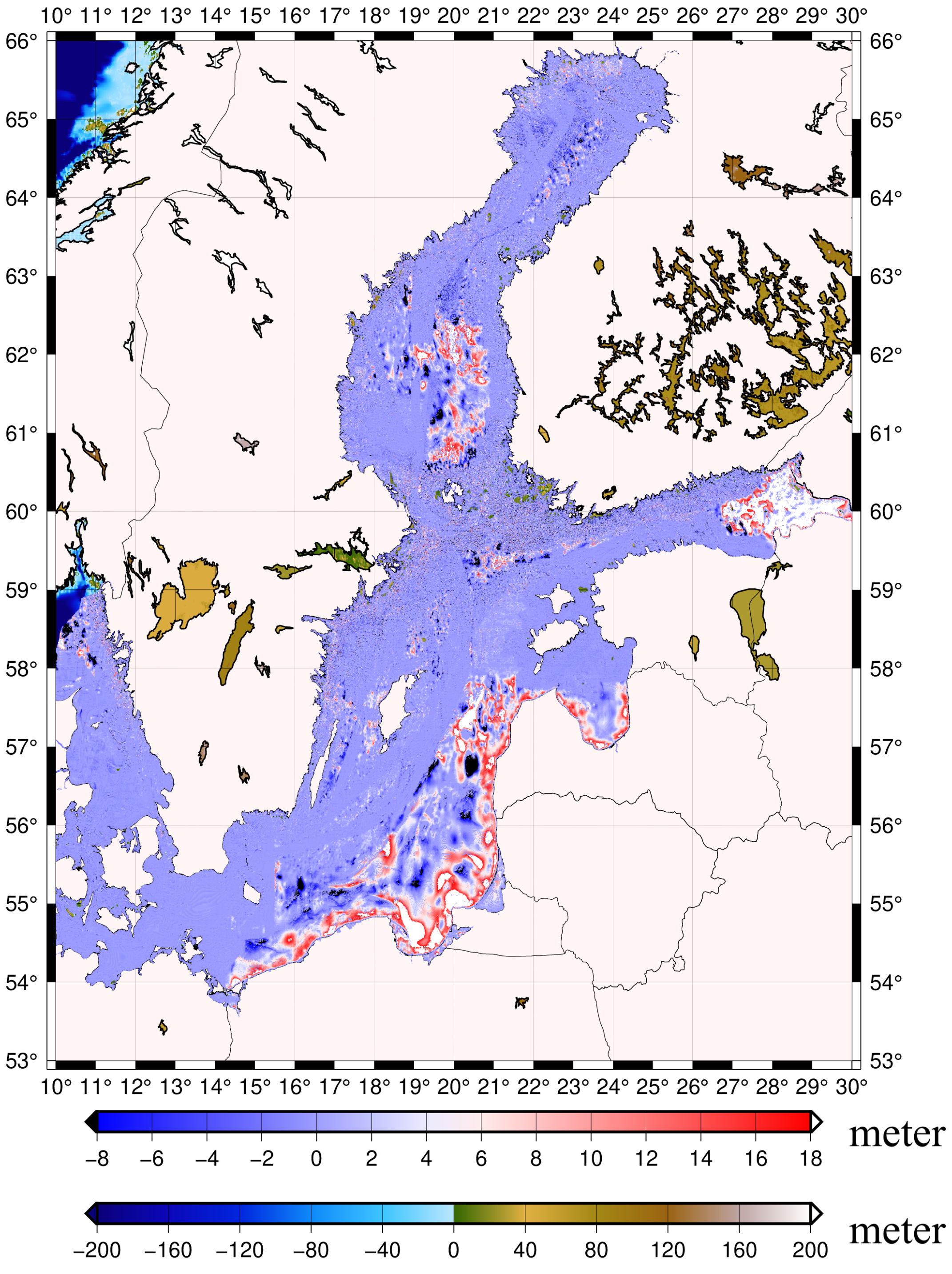

Figure 1: Comparisons of GDEMM2024 with 500 m grid resolution Baltic_Sea_Bathymetry_Database_v091 over the Baltic Sea at 8176191 grid points. For most of the Baltic Sea the two models are in agreement within few meters whereas south-east experience larger differences.

Max: 209.324, Min: -220.449, Mean: 1.572, R.m.s: 7.351, Std: 7.181.
